# Gamma power in rural Pakistani children: Links to executive function and verbal ability

**DOI:** 10.1016/j.dcn.2017.03.007

**Published:** 2017-03-31

**Authors:** Amanda R. Tarullo, Jelena Obradović, Brandon Keehn, Muneera A. Rasheed, Saima Siyal, Charles A. Nelson, Aisha K. Yousafzai

**Affiliations:** a Department of Psychological and Brain Sciences, Boston University, 64 Cummington Mall, Boston, MA 02215, United States; b Graduate School of Education, Stanford University, United States; c Departments of Speech, Language, and Hearing Sciences and Psychological Sciences, Purdue University, United States; d Department of Paediatrics and Child Health, Aga Khan University, Pakistan; e Departments of Pediatrics and Neuroscience, Harvard Medical School, Boston Children’s Hospital, United States; f Harvard Graduate School of Education, United States; g Department of Global Health and Population, T. H. Chan School of Public Health, Harvard University, United States

**Keywords:** EEG, Low- and middle-income countries, Poverty, Executive function, Cognitive development, Sex differences

## Abstract

Children in low- and middle-income countries are at high risk of cognitive deficits due to environmental deprivation that compromises brain development. Despite the high prevalence of unrealized cognitive potential, very little is known about neural correlates of cognition in this population. We assessed resting EEG power and cognitive ability in 105 highly disadvantaged 48-month-old children in rural Pakistan. An increase in EEG power in gamma frequency bands (21–30 Hz and 31–45 Hz) was associated with better executive function. For girls, EEG gamma power also related to higher verbal IQ. This study identifies EEG gamma power as a neural marker of cognitive function in disadvantaged children in low- and middle-income countries. Elevated gamma power may be a particularly important protective factor for girls, who may experience greater deprivation due to gender inequality.

## 1 Introduction

Children in low- and middle-income countries (LMIC) experience numerous co-occurring forms of adversity that are detrimental to cognitive development, including absolute poverty, stunted growth, macro and micronutrient deficiencies, environmental toxins, and inadequate cognitive stimulation (Black et al., 2013, Walker et al., 2011). The global toll of these cumulative risks to cognitive development is staggering: More than 200 million children under age five in LMIC do not attain their cognitive potential (Grantham-McGregor et al., 2007). A third of three- and four-year-old children in LMIC fail to reach basic developmental milestones such as the ability to follow simple directions (McCoy et al., 2016). The early environment shapes brain development (Malter Cohen et al., 2013, Nelson et al., 2011). Since studies of children in high-income countries (HIC) show that early poverty-related adversity inflicts lasting damage on neurodevelopmental processes essential to higher-level cognition (Johnson et al., 2016), unrealized developmental potential in LMIC likely reflects compromised neural development. However, no study has examined neural markers of early childhood cognitive function in a LMIC population. Given that gamma power has well-established links to cognition among young children from HIC (Benasich et al., 2008), it represents a promising neural index to investigate early childhood brain-behavior associations in LMIC.

Gamma activity consists of high frequency oscillations, recorded from the scalp using electroencephalography (EEG), and is thought to underlie temporal integration of sensory input with higher-level cognitive processes (Kaiser and Lutzenberger, 2005). In adults in HIC, gamma increases during tasks that require executive function (EF; Lakatos et al., 2008) and semantic processing (Fitzgibbon et al., 2004).

Gamma activity increases as children develop, with a peak at 4–5 years of age, particularly in frontal regions (Takano and Ogawa, 1998); therefore, higher resting frontal gamma in early childhood may index neural maturity. Gamma reflects synchronization of neuronal firing, which facilitates development of efficient neural networks (Uhlhaas et al., 2010). Thus, increased gamma is also an indicator of ongoing neural processes that are likely to shape developing neural architecture in ways that promote efficient cognitive function (Gou et al., 2011).

In HIC, the gamma-cognition association is present in early childhood. Higher resting frontal and parietal gamma power in newborns predicted 15-month working memory and language comprehension, respectively (Brito et al., 2016). Resting frontal gamma in 16- to 36-month-olds related positively to language, cognition, and inhibitory control (Benasich et al., 2008) and predicted preschool language outcomes (Gou et al., 2011). Thus, gamma power in the frontal region appears particularly important to early childhood cognitive function.

The gamma-cognition association has not been studied among children in LMIC, whose brains are developing in the context of multiple risks. Limited access to prenatal care and to medically attended births increases perinatal complications and neonatal mortality (Katz et al., 2013). Other risks include food insecurity, infectious disease, nutritional deficiencies, inadequate water and sanitation, lack of cognitive stimulation, extreme poverty, maternal depression, exposure to violence, and limited access to education (see for review, Walker et al., 2011). Globally, as of 2011, 165 million children under age 5 showed stunting, as indexed by low height for age, and 52 million were wasting, as indexed by low weight for height (Black et al., 2013). These risks have cumulative effects on cognitive development across early childhood (Hamadani et al., 2014).

If gamma-cognition associations observed in HIC generalize to a severely disadvantaged LMIC, it would suggest a universal and context-independent role for gamma as an index of cognitive function. Because children in LMIC are at risk of cognitive deficits (Grantham-McGregor et al., 2007), it is critical to elucidate mechanisms underlying early cognitive development in LMIC.

In HIC, neural correlates of cognition often differ by gender. Preschool girls and boys had different EEG patterns during an inhibitory control task (Cuevas et al., 2016). Adolescents (Christakou et al., 2009) and adults (Bell et al., 2006) show gender differences in neural activation during EF despite equivalent performance. These gender differences partially reflect sexually dimorphic brain development due to prenatal and perinatal gonadal hormone exposures (Nugent et al., 2012).

Gender disparities in health, knowledge, and standard of living are greater in countries with low to middle human development indices (UNDP, 2014). In LMIC, being female has been associated with lower cognitive performance (Escueta et al., 2014). To the extent that girls have less access to education and receive a smaller share of scant resources, boys and girls in LMIC differ in experiences that shape brain development. Given the complex interplay of hormones and experience, neural organization and gamma-cognition associations may be gender-specific.

### 1.1 Current study

Our goals were (1) to determine if early childhood gamma-cognition associations extend to disadvantaged children in LMIC and (2) to examine whether gamma-cognition relations differ by gender. We were particularly interested in frontal gamma power because gamma – cognition associations reported for this age in HIC are specific to the frontal region (e.g. Gou et al., 2011) and because frontal gamma activity at this age is thought to indicate neural maturity (Benasich et al., 2008, Takano and Ogawa, 1998). We examined EEG, EF, and cognition measures collected from 48-month-old children in rural Pakistan during a follow-up assessment for the Pakistan Early Child Development Scale-Up (PEDS) Trial, a community-based, cluster-randomized controlled trial of early responsive stimulation (RS) and enhanced nutrition (EN) interventions from birth to two years (Yousafzai et al., 2014). Because the RS intervention was linked to better 48-month cognition (Yousafzai et al., 2016), we examined intervention exposure in relation to gamma. We also tested 24-month height-for-age (HAZ; an indicator of stunting); hemoglobin levels (a proxy indicator for anemia); and family wealth as possible covariates.

Participants lived in the Naushero Feroze District in Sindh Province, Pakistan, which is mainly agricultural and highly impoverished. Within Pakistan, the rural poor face disparities in health (Di Cesare et al., 2015) and education (UNICEF, 2014) compared to wealthier urban counterparts. Gender compounds these inequalities, and gender disparities are often more pronounced in rural areas. For example, in Sindh Province, only 31% of rural girls aged 10–12 years attend school, as compared to 62% of rural boys and 81% of urban boys and girls (UNICEF, 2013).

We hypothesized that higher frontal gamma in rural Pakistani children would index better EF, verbal IQ, and performance IQ. Based on gender disparities in early experience in this population, we expected gamma-cognition associations to differ by gender.

## 2 Methods

### 2.1 Participants

The final sample consisted of 105 children (52 girls) who were enrolled in the original PEDS trial from birth to 24 months, participated in the longitudinal follow-up at 48 months (M = 48.24, SD = .24), and had usable EEG. From the re-enrolled sample of 1302, we randomly selected 219 for EEG data collection, stratified by intervention group. Children selected for EEG did not differ from the rest of the re-enrolled sample on 48-month demographic variables or EF. They had slightly higher 48-month verbal IQ (*t*(1238) = 2.17, *p* = .03, mean difference = 1.62) and performance IQ (*t*(1263) = 2.38, *p* = .02, mean difference = 1.66) compared to the rest of the sample. Of these 219 children, 114 did not have sufficient EEG data (see below for inclusion criteria), resulting in a final sample of 105. Children with usable EEG did not differ from children with insufficient EEG on 48-month demographic variables or cognition.

In the final sample of 105 children, 59.0% of mothers and 22.9% of fathers were illiterate, and mothers had minimal formal education (M = 2.42 years, SD = 4.04, range 0–16). Mothers were predominantly housewives (72.4%) and fathers mainly were daily-wage laborers (41.3%) or in agriculture (21.2%). At 48 months, 28.4% of households reported food insecurity.

### 2.2 Procedures

A birth-cohort was enrolled in the PEDS trial from birth to 24 months (Yousafzai et al., 2014). This study uses data collected at 48 months (Yousafzai et al., 2016) by a community-based assessment team. All experimenters were extensively trained in standardized testing protocols as well as in how to build rapport with the children, create a comfortable testing environment, and introduce the EEG and apply the net in a manner that set the children at ease. Each day, three to four children and their families were transported in one vehicle to the testing center for EEG assessment. All children were tested between approximately 10:00 am and 3:00 p.m. Before each child was assessed, the assessor would check if they needed a snack, drink or rest. Once all children were assessed, the families would be transported home in the same vehicle. Assessments were administered in the local language, Sindhi.

### 2.3 Measures

Table 1 reports descriptive statistics.

**Table 1:** Descriptive Statistics.

Cognitive Function	*M (SD)*
Verbal Intelligence (VIQ) N = 102	78.79 (7.87)
Performance Intelligence (PIQ) N = 104	81.28 (9.56)
Executive Function (EF)^a^ N = 98	.01 (.64)

EEG Gamma Power (μV^2^)^b^	*M (SD)*
Frontal 21–30 Hz N = 105	.36 (.20)
Parietal 21–30 Hz N = 105	.26 (.11)
Frontal 31–45 Hz N = 105	.13 (.06)
Parietal 31–45 Hz N = 105	.11 (.06)

*Notes*: ^a^ The EF composite is a standardized average of up to 6 tasks. Included participants had valid scores on at least 3 tasks. ^b^ Raw values for EEG gamma power are reported. These raw values were log transformed prior to analysis to meet the assumptions of normality.

#### 2.3.1 Electrophysiological recording and analysis

EEG was recorded using a 64-channel high-density Geodesic sensor net (Electrical Geodesics, Inc.; Eugene, OR) and a NetAmps 300 high-input amplifier. To decrease attrition, ocular (EOG) electrodes were not used. The net was soaked in electrolyte solution (6cc KCl/liter distilled water) to facilitate electrical contact. Impedances were accepted if lower than 50 KOhm. Data were sampled at 500 Hz and referenced to the vertex (Cz). Eyes-open continuous EEG was recorded for four blocks of one minute each. A central fixation was presented on a gray background. A brief silent video played between blocks.

Data were pre-processed using NetStation 4.5 and EEGLAB (Delorme et al., 2011). A 50 Hz notch-filter was applied, and data was segmented into one-second epochs. Artifact detection involved automatic and hand-editing procedures. Epochs were rejected if they contained blinks, eye movements, drift, or muscle artifact. Bad channels were replaced using spherical spline interpolation, and data were re-referenced to the average reference. Inclusion criteria were <15% bad channels (i.e. fewer than 10 bad channels) and at least 45 epochs of artifact-free data out of a possible 240 epochs. Of the 219 children selected for EEG, reasons for exclusion were no data (4), excessive bad channels (38) and insufficient epochs (72), resulting in a final sample of 105 children with at least 45 s of artifact-free EEG (M = 77.79 s, SD = 25.53 s). Rates of data loss reflect technical difficulties and electrical noise, largely because electricity was only available for half the day.

Regions of interest (ROI) included frontal (6, 12, 60) and parietal (28, 34, 42) channels, corresponding to frontal (Fz, F3, F4) and parietal (Pz, P3, P4) 10–10 locations. The frontal ROI was chosen because resting frontal gamma has been related to early childhood cognitive function in HIC (Benasich et al., 2008, Brito et al., 2016, Gou et al., 2011). The inclusion of a parietal ROI paralleled prior early childhood gamma power research in HIC (Brito et al., 2016, Tomalski et al., 2013) and served as a comparison to determine whether gamma effects were specific to the frontal region. Time-frequency decomposition was performed in one-second epochs using Fast Fourier Transformations. Spectral power was computed in low (21–30 Hz) and high (31–45 Hz) gamma bands, in line with Tomalski et al. (2013). Power was also computed in the 4–6 Hz and 6–9 Hz bands widely used in early childhood research, for purposes of follow-up analyses to ascertain whether findings were specific to the gamma band. Raw power was subjected to natural log transformation.

#### 2.3.2 Executive function

Since there was no existing preschool EF battery validated in a rural, disadvantaged LMIC context, an extensive adaptation process was completed to arrive at six tasks judged developmentally and culturally appropriate (Obradović et al., 2016a, Obradović et al., 2016b). Descriptive statistics provided below for each task are for those participants who passed the practice items for that task. Four tasks assessed inhibitory control, the ability to suppress a dominant motoric or verbal response. During the *Shape Stroop* task (Carlson, 2005), children were asked to point to a small fruit embedded within a different larger fruit (e.g., a small apple inside a large banana) and suppress the potent response to choose the large, more salient fruit (e.g., large apple). The total score reflected the percent correct across 3 test trials (*M* = 52.1%, *SD* = 36.6%). During a variant of the *Knock-Tap* task (Luria, 1966) widely used with preschoolers (Blair, 2003, Diamond and Taylor, 1996), children were asked to tap on the table with their hand after the assessor knocked on it, and, conversely, to knock after the assessor tapped. The total score reflected the percent correct across 16 test trials (*M* = 49.3%, *SD* = 24.9%). During the *Reverse Categorization* task (Carlson, 2005), children were asked to say “little” when presented with a picture of a big cat and to say “big” when presented with a picture of a little cat. The total score reflected the percent correct across 16 test trials (*M* = 54.3%, *SD* = 30.6%). During the *Go/NoGo* task (Willoughby et al., 2010), children were asked to press a desk bell when presented with an image of a cat (go stimulus) and not to press the bell when presented with an image of a dog (no-go stimulus). The total score (*M* = 79.5%, *SD* = 23.5%) reflected the percent accuracy on seven “no-go” trials for children who demonstrated at least 76% accuracy on 17 “go” trials. During the *Forward Word Span*, a working memory task, children were asked to repeat verbally presented word sequences of increasing length. The total score (*M* = 1.62, *SD* = 0.70) represented the longest span for which at least two test trials were repeated correctly, plus 0.5 if one longer sequence was correctly repeated at the next level (Noël, 2009). Children who could not repeat any words, or only one word, were given a score of 1. The *Separated Dimensional Change Card Sort task* (S-DCCS; Diamond et al., 2005) was developed to measure preschoolers’ ability to switch attention between two different dimensions, using a set of colored cards (green or yellow) featuring the black silhouette of a common shape (star or truck). Children were asked to complete six color trials, and then, after a rule switch, six shape trials. This differs from the standard DCCS in that the background color and the shape are two separate sources of information, which make it easier for young children to inhibit the pre-switch rule as compared to the standard DCCS, in which color is a property of the shapes themselves (Diamond et al., 2005). The total score reflected the percentage of correct post-switch trials (*M* = 65.4%, *SD* = 35.6%).

A composite was created by averaging valid scores across six tasks (Cronbach’s α = 0.60, *M* = 0.01, *SD* = 0.64). Based on research demonstrating that a three-task battery is acceptable to measure children’s overall EF (Willoughby et al., 2013), the EF composite included scores for children who passed comprehension criteria for three or more tasks (93% of children in the current sample; *M* *=* 5.36 tasks, *SD* = 1.05).

#### 2.3.3 General cognitive ability

The Wechsler Preschool and Primary Scale of Intelligence – III (WPPSI-III; Wechsler, 2002) assessed 48-month verbal and nonverbal ability. As with the EF battery, the WPPSI-III went through extensive cultural adaptation (see Rasheed et al., 2016). Scale scores from Information, Vocabulary, and Word Reasoning subtests were combined into a Verbal Intelligence Composite (VIQ; Cronbach’s α = .88, *M* = 78.79, *SD* = 7.87). Scale scores from Block Design, Matrix Reasoning, and Picture Concepts subtests were combined into a Performance Intelligence Composite (PIQ; Cronbach’s α = .69, *M* = 81.28, *SD* = 9.56). VIQ and PIQ were considered separately rather than using Full Scale IQ to test for possible differential associations with gamma.

#### 2.3.4 Intervention exposure

The PEDS Trial was a cluster-randomized, controlled trial with a 2 × 2 factorial design (Yousafzai et al., 2014). The RS intervention promoted sensitive and responsive caregiving with child stimulation using the adapted United Nations Children’s Fund and World Health Organization’s Care for Child Development curriculum (UNICEF, 2011), in which community health workers provided coaching and support during monthly home visits and parenting groups. The EN intervention focused on expanding nutrition education provided by community health workers and, for children more than six months old, delivery of multiple micronutrient powder. Dummy variables represented exposure to RS (N = 43) and EN (N = 51). RS exposure related to higher EF, VIQ, and PIQ in the overall cohort (Yousafzai et al., 2016) and to higher EF in the current subsample (*p* = .01); thus, we tested intervention status as a possible covariate.

#### 2.3.5 Covariates

Height at 24 months was converted with WHO Anthro software V3.2.2 to a standardized *HAZ index*, used as a continuous measure (*M* = −2.06, *SD* = 1.13, range = −5.41–.65). At 24 months, 55.3% of children were stunted, defined as HAZ more than 2 SDs below the WHO mean. *Hemoglobin levels* were assessed from a 24-month blood draw (*M* = 9.29 g/dl, *SD* = 1.55, range = 6–13), and used as a continuous measure. At 24 months, 86.1% of children met criteria for anemia, defined as a hemoglobin level below 11 g/dl. *Family wealth* was assessed at 24 months by caregiver report reflecting household assets (e.g. television) and dwelling characteristics (e.g. access to water). Principle components analysis generated a standardized factor score representing family wealth (*M* = .26, *SD* = .93, range = −1.32–2.75). *Head Circumference* was assessed at 48 months (*M* = 48.07 cm, *SD* = 1.60 cm).

### 2.4 Data analysis plan

We tested our hypotheses using Repeated Measures GLM in SPSS. We used Repeated Measures Analyses of Covariance (RM-ANCOVAs) because EEG power is highly correlated across regions and frequencies. For all RM-ANCOVAs, gamma power was the dependent repeated measure, and within-subjects factors were band (21–30 Hz; 31–45 Hz) and region (frontal; parietal). We used Greenhouse-Geisser corrections when the sphericity assumption was violated. Interactions were interpreted using estimated marginal means.

Potential covariates were tested using RM-ANCOVAs. First, number of bad channels and number of artifact-free epochs were tested, because EEG power can be affected by data quality. Second, because absolute EEG power can be affected by anatomical factors, we tested association with 48-month head circumference. Third, to test for intervention effects on gamma, we examined exposure to RS and EN interventions as between-subjects factors. Fourth, to determine whether variation in deprivation at the conclusion of the PEDS trial related to gamma, we tested the association of gamma with 24-month HAZ, hemoglobin levels, and family wealth in separate RM-ANCOVAs. Finally, to test whether frontal gamma is associated with cognition and whether gamma-cognition associations differed by gender, we conducted three RM-ANCOVAs. For each model, we entered the continuous cognitive variable of interest (EF, VIQ, & PIQ) as a continuous covariate, and gender as a between-subjects factor (Hoffman, 2014, Sweet and Grace-Martin, 2011). This approach enabled us to test main effects of continuous cognitive variables in relation to gamma as well as interactions of continuous cognitive variables with within-subjects factors (band, region) and the between-subjects factor (gender).

While our hypotheses focused on gamma power based on prior literature, we conducted post-hoc analyses repeating each model for the 4–6 Hz and 6–9 Hz bands, which are frequency bands widely used in early childhood research, in order to determine specificity of results to the gamma band.

## 3 Results

### 3.1 Preliminary analyses

There were no main or interactive effects of number of artifact-free EEG epochs, 48-month head circumference, RS or EN intervention exposure, 24-month HAZ, 24-month hemoglobin levels, or 24-month family wealth on gamma. Thus, it was unnecessary to control for these measures in the main analyses. There was a main effect of the number of bad channels on gamma, *F*(1, 103) = 7.18, *p* = .009, such that gamma was higher when there were more bad channels. There was also a band by number of bad channels interaction, such that the effect of bad channels on gamma was more pronounced in the 31–45 Hz band, *F* (1, 103) = 20.99, *p <* .001. Thus, number of bad channels was co-varied in all subsequent analyses.

### 3.2 Gamma power and cognition

#### 3.2.1 Executive function

**Fig. 1:**
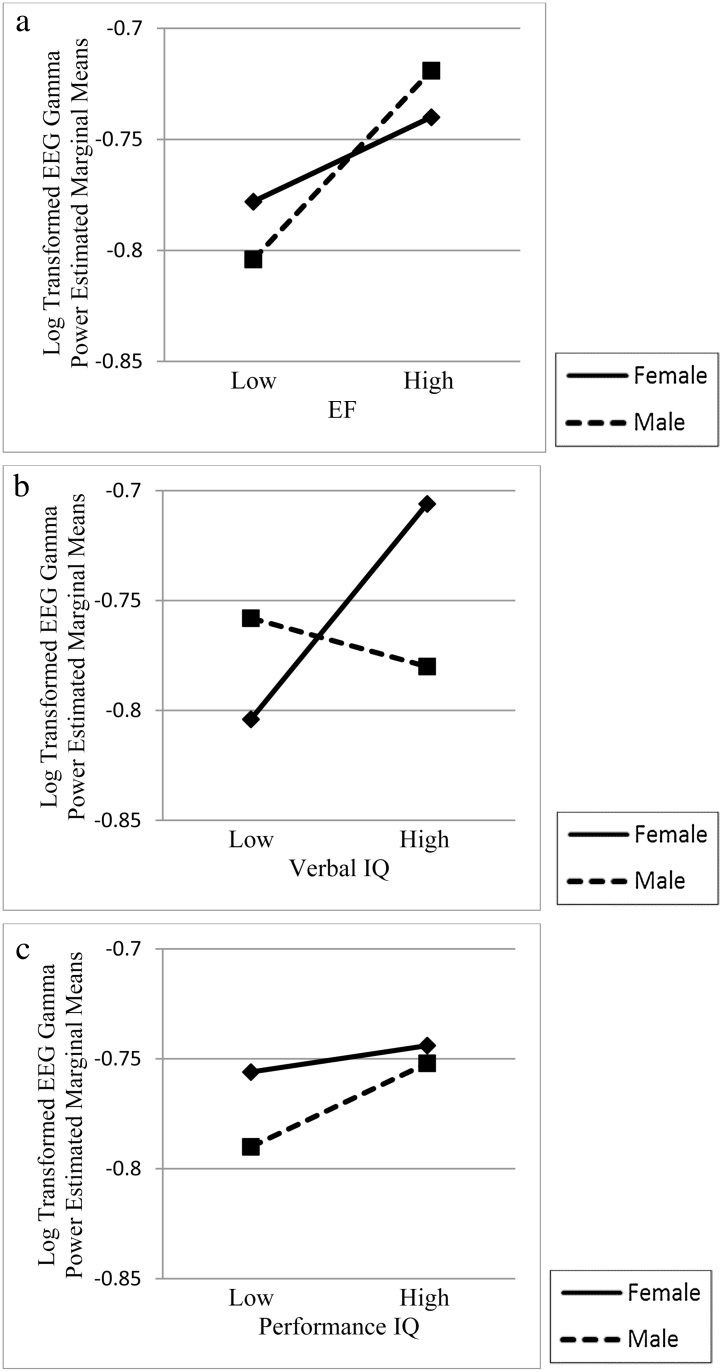
Estimated marginal means for log transformed gamma power by gender as a function of executive function (EF, 1a), Verbal IQ (VIQ, 1b) and Performance IQ (PIQ, 1c) scores 1 SD below and 1 SD above the mean. Gamma power values are negative due to log transformation of a value less than 1. In Fig. 1a, children with high EF have higher gamma, regardless of gender. In Fig. 1b, higher VIQ related to higher gamma for girls only. In Fig. 1c, gamma and PIQ are unrelated.

There was a main effect of EF on gamma, *F*(1, 93) = 4.54, *p* = .036, partial η^2^ = .05. Parameter estimates indicated that children who scored higher on EF showed increased gamma power. There was no main effect of gender, and there were no interaction effects, indicating that the association of better EF with higher gamma was consistent across gender, bands, and regions. Fig. 1a depicts the estimated marginal means for gamma power by gender for EF scores 1 SD below the mean (low EF) and 1 SD above the mean (high EF).

#### 3.2.2 VIQ

We found a VIQ × gender interaction, *F*(1, 97) = 4.71, *p* = .033, partial η^2^ = .05. Estimated marginal means indicated that higher VIQ related to higher gamma for girls only. Fig. 1b shows the estimated marginal means for gamma by gender for VIQ scores 1 SD below and above the mean. We graphed VIQ at these discrete points to illustrate and interpret the VIQ × gender interaction. There was a main effect of gender, *F*(1, 97) = 4.46, *p* = .037, partial η^2^ = .04 but no main effect of VIQ. There were no interactions with band or region.

#### 3.2.3 PIQ

There were no main or interactive effects of PIQ or gender on gamma. Fig. 1c shows estimated marginal means for gamma by gender for PIQ 1 SD below and above the mean.

### 3.3 Follow-up analyses

Frontal and parietal 4–6 Hz and 6–9 Hz power were not associated with any of the cognitive measures or with gender. Thus, of the bands examined, results were specific to the gamma bands, consistent with our hypotheses.

## 4 Discussion

This is the first study to demonstrate links between gamma and cognition in a LMIC context. Higher resting EEG gamma power, reflecting more high frequency neural oscillations, was linked to higher EF and verbal ability in 48-month-old children in rural Pakistan. The association between gamma and verbal ability was significant only for girls. Worldwide, millions of young children in LMIC fail to realize their cognitive potential (Grantham-McGregor et al., 2007), yet the neural mechanisms underlying these developmental failures have gone unstudied. The current findings move the field forward by establishing associations between brain activity and cognition in young children in a rural LMIC.

Previous early childhood research in the U.S. reported strikingly similar associations of gamma power with executive function and language ability (Benasich et al., 2008, Brito et al., 2016, Gou et al., 2011). Results are also in line with an extensive literature associating gamma activity with executive function and language in adults in HIC (e.g. Lakatos et al., 2008). Uhlhaas et al. (2010) argued that synchronization of gamma oscillations promotes the development of efficient neural circuits. Our results extend this association to severely disadvantaged children in a rural LMIC, supporting the idea that the role of gamma oscillations in promoting brain development and facilitating higher-level cognition may be universal rather than specific to privileged environments. Thus, the current study can inform theoretical and empirical work on gamma activity.

Contrary to our hypotheses, gamma-cognition associations in the current sample were not specific to the frontal region, as has been observed for children of a similar age in HIC (Gou et al., 2011). Parietal gamma was included in our models as a comparison, and all effects were observed across frontal and parietal regions alike. Takano and Ogawa (1998) describe a developmental peak in gamma activity at age 4–5 years, particularly in the frontal region. However, the developmental trajectory of gamma development in disadvantaged LMIC children is unknown. It could be that their brains are less mature and more diffuse in their activation, such that the association of gamma power with cognition is not yet specific to the frontal region at age 4 years. In that case, one would expect to find specific associations with frontal gamma emerging in older LMIC children. In a HIC study, newborn gamma in the parietal region predicted later auditory comprehension (Brito et al., 2016), supporting the possibility that gamma-cognition associations may not be limited to the frontal region. More research is needed assessing trajectories of early childhood gamma development across cultures and across both advantaged and disadvantaged contexts.

Higher gamma power was concurrently related to better EF performance in the current sample, as assessed by a battery of executive function tasks adapted to be culturally and developmentally appropriate (Obradović et al., 2016a). Early childhood research in HIC has related gamma power to several specific domains of executive function. Brito et al. (2016) reported that newborn frontal gamma power predicted working memory ability at 15 months of age. Benasich et al. (2008) found that frontal gamma power at 24 months was associated with concurrent parent-reported inhibitory control and attention shifting. The battery in the current study encompassed all of these domains: it included inhibitory control tasks, a working memory task, and an attention shifting task. The efficient neural processing reflected by higher gamma power may facilitate emerging EF abilities.

In contrast to EF results, the association of gamma and verbal ability was moderated by gender. Girls with higher gamma had higher verbal ability, but for boys, gamma and verbal ability were unrelated. This raises the question of why we observed gender specificity for verbal ability but not for EF. These domains differ both in the neural networks involved and in developmental timing. EF is just emerging in the preschool years (Zelazo et al., 2003). Because maturation of brain regions such as the prefrontal cortex is a prerequisite for EF (Zelazo et al., 2003), gamma at this age may identify children with sufficient neural maturity to enable EF. Verbal abilities develop on an earlier timetable, particularly in girls (Fenson et al., 1994), and the moderating role of gender may be more evident in a more established cognitive domain. Future research should clarify whether gender differences in brain-behavior associations in LMIC vary across development.

There are several possible reasons why we observed a gender interaction in the association of gamma and verbal ability when prior research in HIC did not. First, children in the current study were 48 months when gamma was measured, whereas prior studies measured gamma in newborns (Brito et al., 2016) or toddlers (Gou et al., 2011). Perhaps the differential association of gamma and verbal ability reflects emerging sexual dimorphisms in neural organization that are not evident until the preschool years.

Second, the gender interaction may reflect differential *experience* of girls and boys in Pakistan, where females have less access to education and resources (UNDP, 2014). The neurophysiological underpinnings associated with gamma may be a particularly important protective factor for girls in a LMIC context in which they experience greater deprivation. If that explanation is correct, gender differences in gamma-cognition associations should only be evident in countries with marked childhood gender disparities. A third possibility is that boys are neurodevelopmentally vulnerable. In LMIC, they have higher rates of stunting and early childhood mortality (Black et al., 2013). Perhaps one aspect of their neurodevelopmental sensitivity to risk is loss of the gamma-language association under conditions of high adversity. Girls’ brains may be more robust to risk, such that, even in adverse contexts, they still capitalize on gamma oscillations to develop better verbal ability. If that explanation is correct, then association of gamma and verbal ability should be limited to girls in all LMIC high-risk contexts, regardless of gender disparities in experience.

Distinguishing among these alternate hypotheses for the interaction of gender and verbal ability will require replicating the current study in different LMIC contexts that include both high and low adversity and high and low gender inequities in development, as quantified by the Human Development Index and Gender Development Index (UNDP, 2014). Such research will be crucial to establish the extent to which gender-specific brain-cognition associations reflect biologically determined sex differences in neural organization, societally determined gender differences in experience, and/or sex differences in neural sensitivity to adverse experiences.

Children in this study were part of a birth cohort who had participated in the PEDS trial, a cluster-randomized controlled trial of responsive stimulation and enhanced nutrition interventions (Yousafzai et al., 2014). Although both interventions have been shown to have positive effects on cognitive outcomes (Obradović et al., 2016b, Yousafzai et al., 2014, Yousafzai et al., 2016), neither of the interventions related to gamma. Perhaps effects of environmental enrichment on gamma were too small to detect, or worked through changes in parenting targeted by the intervention. Two-year-old HAZ, hemoglobin levels, and family wealth did not predict gamma. The entire sample was highly disadvantaged, so there was a constrained range for these measures.

Our findings have several implications from a global health perspective. First, results suggest that gamma power may serve as a useful early biomarker for identifying which children within a disadvantaged LMIC context are at greatest risk of not reaching their cognitive potential. One in three children in LMIC do not meet basic developmental milestones due to extreme poverty, stunting, and environmental deprivation (McCoy et al., 2016). Better approaches to targeting intervention efforts are needed urgently. The current analyses were cross-sectional. However, given prior research in which gamma activity predicted cognitive outcomes (Brito et al., 2016, Gou et al., 2011), gamma among children in LMIC may well predict trajectories of cognitive development.

Second, gamma power may be of particular value for assessing children in LMIC because existing behavioral measures of cognition have been used predominantly in HIC and may not be appropriate to children’s range of experiences. An extensive process of adaptation and validation is required for each cultural context, as was completed for the executive function and IQ measures in this study (Obradović et al., 2016a, Obradović et al., 2016b, Rasheed et al., 2016). In the absence of validated behavioral measures, resting gamma power may provide an unbiased neural index of cognitive function.

### 4.1 Limitations

This study is groundbreaking in examining neural correlates of cognitive function among disadvantaged children in rural LMIC. However, there are limitations. First, about half the children for whom EEG was recorded had insufficient data. Children with and without usable EEG did not differ on demographic or cognitive measures. Thus, missing data likely was due to technical challenges of EEG recording in this context, which rarely has been attempted. Future studies should refine procedures to optimize data quality in rural LMIC. Also, because we relied on an existing birth cohort in rural Pakistan, the study did not include a comparison group of wealthier urban children in Pakistan. The study was not designed to compare groups with lower and higher adversity, but rather to test gamma as a neural marker of individual differences in cognition *within* a highly disadvantaged, rural LMIC context. Future research should examine brain-cognition associations in a variety of LMIC contexts.

### 4.2 Conclusions

This study advances our understanding of mechanisms underlying cognitive development within LMIC. Rural Pakistan typifies adverse conditions experienced by disadvantaged children in LMIC, so the association of gamma with EF and verbal ability in this context strongly indicates that resting gamma is a valid marker of early childhood cognition and neural maturity in other LMIC contexts. The gender-specific association of gamma and verbal ability highlights the need to consider both biologically mediated sex differences in neural organization and socially mediated gender disparities in experience to elucidate further the neural mechanisms of cognitive development in LMIC.

## Conflict of Interest

None.
